# Can Radiomics Provide Additional Diagnostic Value for Identifying Adrenal Lipid-Poor Adenomas From Non-Adenomas on Unenhanced CT?

**DOI:** 10.3389/fonc.2022.888778

**Published:** 2022-04-29

**Authors:** Binhao Zhang, Huangqi Zhang, Xin Li, Shengze Jin, Jiawen Yang, Wenting Pan, Xue Dong, Jin Chen, Wenbin Ji

**Affiliations:** ^1^ Department of Radiology, Taizhou Hospital of Zhejiang Province affiliated to Wenzhou Medical University, Taizhou, China; ^2^ Department of Radiology, Taizhou Hospital of Zhejiang Province, Shaoxing University, Taizhou, China; ^3^ Department of Radiology, Taizhou Hospital, Zhejiang University, Taizhou, China

**Keywords:** adrenal adenoma, adrenal gland neoplasms, computed tomography, diagnosis, radiomics

## Abstract

**Background:**

It is difficult for radiologists to differentiate adrenal lipid-poor adenomas from non-adenomas; nevertheless, this differentiation is important as the clinical interventions required are different for adrenal lipid-poor adenomas and non-adenomas.

**Purpose:**

To develop an unenhanced computed tomography (CT)-based radiomics model for identifying adrenal lipid-poor adenomas to assist in clinical decision-making.

**Materials and methods:**

Patients with adrenal lesions who underwent CT between January 2015 and August 2021 were retrospectively recruited from two independent institutions. Patients from institution 1 were randomly divided into training and test sets, while those from institution 2 were used as the external validation set. The unenhanced attenuation and tumor diameter were measured to build a conventional model. Radiomics features were extracted from unenhanced CT images, and selected features were used to build a radiomics model. A nomogram model combining the conventional and radiomic features was also constructed. All the models were developed in the training set and validated in the test and external validation sets. The diagnostic performance of the models for identifying adrenal lipid-poor adenomas was compared.

**Results:**

A total of 292 patients with 141 adrenal lipid-poor adenomas and 151 non-adenomas were analyzed. Patients with adrenal lipid-poor adenomas tend to have lower unenhanced attenuation and smoother image textures. In the training set, the areas under the curve of the conventional, radiomic, and nomogram models were 0.94, 0.93, and 0.96, respectively. There was no difference in diagnostic performance between the conventional and nomogram models in all datasets (all p < 0.05).

**Conclusions:**

Our unenhanced CT-based nomogram model could effectively distinguish adrenal lipid-poor adenomas. The diagnostic power of conventional unenhanced CT imaging features may be underestimated, and further exploration is worthy.

## Introduction

With the marked escalation in the use of diagnostic imaging, incidental detection of adrenal nodules is increasing (1, 2). Benign adrenal adenomas account for 75%−80% of non-tumor cases (3–5). Adrenal adenomas do not require further investigation or clinical intervention, but suspected malignant adrenal tumors (i.e., pheochromocytoma, metastatic tumor) usually require imaging follow-up to determine subsequent treatments, such as adrenalectomy (5–9). Frequently, adrenal adenomas containing abundant intracytoplasmic lipids can be readily diagnosed with high specificity on unenhanced computed tomography (CT) imaging, whereas approximately 30% of adrenal adenomas are lipid-poor, measuring > 10 Hounsfield Unit (HU) on CT (4, 10, 11). Distinguishing adrenal lipid-poor adenomas from non-adenomas is a challenge for radiologists, particularly in cases with an unknown history of primary malignancy, because of the overlap in unenhanced attenuation between them (12–14). Previous studies have suggested that contrast-enhanced washout CT can effectively identify lipid-poor adenomas; however, some hypervascular tumors (i.e., adrenal metastasis of liver cancer) manifest similar washout characteristics, making diagnosis difficult. Chemical-shift magnetic resonance imaging (MRI) is also useful in characterizing adrenal adenomas, but its sensitivity for those measuring more than 30 HU is poor (15). Dual-energy CT can provide energy-spectrum information, which is helpful in distinguishing lipid-poor adenoma; however, lipid-rich adenoma may be misdiagnosed (16, 17).

Radiomics, with features extracted from medical images (i.e., CT and MRI) can produce accurate and robust evidence to assist in clinical decision-making. Radiomics can use high-throughput methods to extract and analyze quantitative information that cannot be assessed by visual inspection of CT, as well as other clinical images, based on intensity, shape, size, and texture (18). Previous studies have shown that texture analysis can be used to distinguish lipid-poor adenomas with high accuracy (14, 19). Therefore, these quantitative radiomics features may be helpful in effectively identifying lipid-poor adenomas.

The purpose of our study was to assess the performance of radiomics in the identification of adrenal lipid-poor adenomas and to develop a diagnostic model to assist in clinical decision-making.

## Materials and Methods

### Study Patients

This retrospective study analyzed consecutive patients admitted to two independent institutions between January 2015 and August 2021, who met the following inclusion criteria: adult patients (aged ≥18 years) with adrenal lesions, who underwent adrenal or abdominal unenhanced and contrast-enhanced CT.

We excluded lesions showing unenhanced attenuation of ≤ 10 HU or macroscopic fat (i.e., lipid-rich adenomas and myelolipomas, respectively); lesions without a solid component, defined as a change in pre- and post-contrast imaging of > 10 HU (i.e., cysts or hematomas); cases without an adequate reference standard, namely adrenal lesions exhibiting an increase of 10%–30% in maximum diameter; lesions with prior systemic or focal therapy; lesions < 10 mm in maximum diameter, which was our cut-off value to avoid partial volume effects; and cases with poor image quality, such as severe motion artifacts.

Institution-based sampling was applied. Cases from institution 1, which had a large number of patients, was randomly divided into a training set and a test set at a ratio of 7:3, while cases from institution 2 were used as the external validation set.

This retrospective study was approved by the Institutional Review Board of institutions 1 and 2, and the requirement for obtaining written informed consent was waived.

### Reference Standard

The final diagnosis of all analyzed lesions was established based on histopathological findings or widely accepted imaging criteria. Lipid-poor adenomas were diagnosed based on histopathology or size stability (< 10% transverse diameter) for at least 12 months during the imaging follow-up. Lesions that exhibited abnormal ^18^F-fluorodeoxyglucose (FDG) uptake, but which satisfied the adenoma criteria, were diagnosed as adenomas. Adrenal lesions in patients with extra-adrenal malignancies were classified as metastases based on at least one of the following criteria: pathologic diagnosis; newly developed or increase in size (at least 30% increase in maximum diameter) within 12 months during the imaging follow-up, and/or interval regression in size following systemic chemotherapy; or abnormal ^18^F-FDG uptake (defined as avid uptake relative to liver parenchyma). The diagnoses of pheochromocytomas and adrenocortical carcinomas were histopathologically confirmed, whereas hematomas and cysts were diagnosed based on typical CT findings and interval follow-up.

For histopathologically confirmed lesions, preoperative unenhanced and contrast-enhanced CT images were used for analysis, whereas for those confirmed by imaging follow-up, the earliest CT images were analyzed.

### Image Acquisition

All unenhanced and contrast-enhanced CT images from the two institutions were obtained using multislice spiral CT scanners (uCT 530; United Imaging, Shanghai, China; Discovery CT750 HD or BrightSpeed 16; GE Healthcare, Chicago, IL, USA). The imaging protocols are described in the Appendix. The image was reconstructed to 2.5-mm, or 5-mm thickness using a standard algorithm and was then uploaded to the image archiving and communication system (PACS).

### Image Analysis

When a patient had multiple adrenal lesions, only the largest (measured maximum diameter) lesion was analyzed to reduce the clustering effect. Two radiologists (reader 1 and reader 2, with 5 and 7 years of experience in abdominal imaging, respectively), who were blinded to the clinical and histopathological information, independently evaluated the conventional CT image features, including maximum tumor diameter, unenhanced attenuation, and lesion distribution. The maximum tumor diameter and unenhanced attenuation were measured manually on slices with the largest lesion area. Unenhanced attenuation was measured using as large a circular or elliptical region-of-interest (ROI) as possible while avoiding lesion margins, normal adrenal parenchyma, calcification, artifacts, and apparent necrotic or cystic areas. The ROI was determined on the contrast-enhanced image and was then copied onto the unenhanced image. Manual correction was performed, if necessary. All measurements were taken twice in one reading session, and the average values were acquired. The two radiologists also measured 37 samples that were randomly selected 1 month later, to assess the reproducibility of assessing the conventional CT features.

### Image Segmentation

The radiomics process included image segmentation, feature extraction, feature selection, and model building.

When a patient had multiple adrenal lesions, only the largest lesion (measured as the maximum diameter) was analyzed. 3D segmentation of the lesions was performed by a radiologist (reader 1), semi-automatically, using the open-source software ITK-SNAP (V3.6.0, http://www.itksnap.org). The most inferior and superior slices were excluded to minimize the effects of partial volume. The same 37 samples were selected to assess the reproducibility of the radiomics features. To test for interobserver and intraobserver reproducibility, two radiologists (reader 3, with 3 years of experience in abdominal imaging, and reader 1) segmented the images again after 1 month. All radiologists were blinded to the patients’ clinical or histopathological information.

### Feature Extraction and Selection

Radiomic features were extracted and filtered from segmented ROIs using Pyradiomics (V3.0.1; Harvard Medical School; https://github.com/Radiomics/pyradiomics), an open-source Python package. Image standardization was not applied when setting the parameters on the Pyradiomics package because CT values reflect real-world values and should be comparable across different scanners.

Thereafter, the graphics were resampled to a pixel space of 1 × 1 × 1 to standardize the 3D-voxel space. The bin-width was set to 3 for discrete voxel intensity to reduce image noise and normalize the image intensity. The VoxelArrayShift was set to 450, which not only prevented negative values, but also limited the volume confounding effect. The details of the Pyradiomics setting parameters are shown in the Appendix.

Radiomic features are mainly divided into three categories: first-order features, shape features, and texture features. These extracted features were in line with the feature definitions described by the imaging biomarker standardization initiative (20). In this study, we did not use image filters. One hundred features were analyzed.

In the process of feature selection, features with interobserver or intraobserver consistency of < 0.8 were excluded. Then, the remaining features with a correlation coefficient > 0.5 between the number of voxels were further excluded. To distinguish lipid-poor adenomas from non-adenomas, variables with no statistically significant difference in the univariate analysis were excluded. The minimal redundancy maximum relevance (mRMR) algorithm was used for initial feature selection, and 10 features were kept. Then the LASSO algorithm, which was suitable for the regression of high-dimensional data, was applied to select significantly distinguishable feature-based minimum binomial deviance by adjusting the penalty coefficient (λ) to construct the radiomic signature with 10-fold cross-validation (21). The radiomic scores (rad-scores) were then calculated by summing the selected features, weighted by the corresponding LASSO coefficients.

### Model Establishment

All the models were built on the training set and validated on the test set, and the external validation set was used for further validation. Univariate logistic regression was used to select conventional features that were risk factors for lipid-poor adenomas. Multicollinearity of the variables was tested using the variance inflation factor (VIF). Next, the conventional features with p < 0.05 and VIF ≤ 5 were introduced into a multivariate logistic regression to build a conventional model, with the minimum Akaike’s information criterion as the stopping rule (22). The radiomics and nomogram models were constructed in the same way as described above, with variations in the variables used. The nomogram model considered both conventional and radiomic features.

### Statistical Analysis

All statistical analyses were performed using SPSS (version 26.0; IBM Corp., Armonk, NY, USA) and R software (version 4.1.0; https://www.Rproject.org). Data consistent with normal distribution were represented by means and standard deviations. Student’s t-test was used to compare the difference between adrenal lipid-poor adenomas and non-adenomas, as well as the difference between the training and test sets. Data that were not normally distributed are shown as median (interquartile interval) values and were compared using the Mann–Whitney U test. Categorical variables were expressed as the number of cases (percentage), and the chi-squared test or Fisher’s exact test was performed. Correlation was evaluated using Spearman’s coefficient. The Hosmer–Lemeshow test was used to evaluate the goodness of fit of the model. The area under the receiver operating characteristic (ROC) curve (AUC) for all datasets was used to evaluate the performance of the model in identifying adrenal lipid-poor adenomas. The cutoff value was set using the maximum Youden index. The differences between ROCs were compared using the DeLong test. The reproducibility of the radiomics features was evaluated using intra-observer correlation coefficients with a two-way random model and absolute type. Statistical significance was set at p < 0.05.

## Results

### Demographic Characteristics

**Figure 1:**
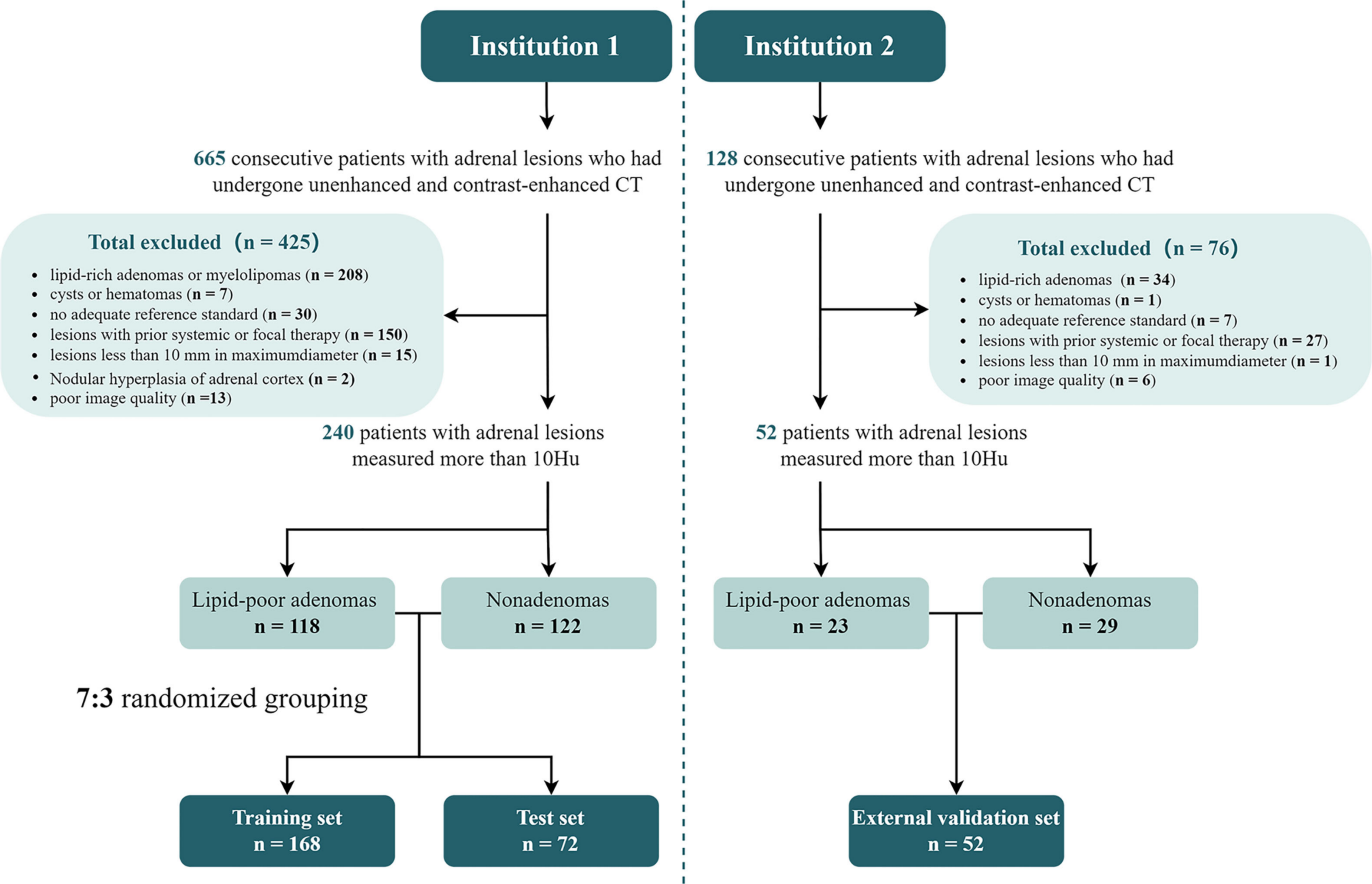
Flowchart of sample inclusion and exclusion in this study.

Overall, 793 adult patients (institution 1: n = 665, institution 2: n = 128) with adrenal lesions who underwent adrenal or abdominal unenhanced and contrast-enhanced CT were admitted to the two institutions. After excluding 501 patients for various reasons (details shown in **Figure 1**) , 292 patients were included in the final analysis.

In institution 1, 118 lesions were diagnosed as lipid-poor adrenal adenomas by the following methods: histopathological diagnosis (n = 108), size stability (n = 9), and abnormal ^18^F-FDG uptake, but meeting the criteria for adenoma (n = 1). The 122 non-adenomas included metastatic tumors (n = 60), hemangiomas (n = 2), spindle cell tumors (n = 1), pheochromocytoma (n = 41), lymphoma (n = 5), adrenocortical carcinoma (n = 6), ganglioneuroma (n = 6), and vascular tumors (n = 1). The diagnostic methods of metastases included pathological diagnosis (n = 8), volume increase newly found or within 12 months of follow-up (n = 22), volume reduction in the interval after systemic chemotherapy (n = 18), and abnormally high ^18^F-FDG uptake (n = 12). All non-adenomas, except for metastases, were confirmed by histopathology.

In institution 2, 23 lesions were diagnosed as lipid-poor adrenal adenomas by histopathological diagnosis (n = 19) or size stability (n = 4). The 29 non-adenomas included metastatic tumors (n = 13), spindle cell tumors (n = 1), pheochromocytoma (n = 11), adrenocortical carcinoma (n = 2), and ganglioneuroma (n = 2). The diagnostic methods of metastases included volume increase either newly found or within the 12-months follow-up (n = 7), volume reduction in the interval after systemic chemotherapy (n = 2), and abnormally high ^18^F-FDG uptake (n = 4). Spindle cell tumors, pheochromocytomas, adrenocortical carcinomas, and ganglioneuromas were confirmed by histopathology.

The basic demographic characteristics of institution 1 are shown in **Table 1**. Lipid-poor adenoma patients tended to be female (64.3%), younger (51.1 ± 11.5) vs 57.1 ± 11.8, p < 0.001), and have higher body mass index (24.5 ± 2.7 kg/m^2^ vs 23.4 ± 3.5 kg/m^2^, p < 0.001). There was no significant difference between the training and test sets for all variables (**Table S1** in appendix). The comparison of demographic and imaging characteristics between institution 1 and 2 are shown in **Table 2**. There was no significant difference between institution 1 and 2 in all variables. Multivariable logistic regression analysis revealed that sex and age were independent predictors of lipid-poor adenomas (**Table 3**).

**Table 1:** Clinical and imaging characteristics of Institution 1.

Variable	Training set			Test set		
	Adrenal nonadenomas (n=84)	Lipid-poor adrenal adenomas (n=84)	p-value	Adrenal nonadenomas (n=38)	Lipid-poor adrenal adenomas (n=34)	p-value
Gender (%)						0.23
female	21 (25.0)	54 (64.3)	< 0.001	17 (44.7)	21 (61.8)	
male	63 (75.0)	30 (35.7)		21 (55.3)	13 (38.2)	
Age*	57.1 ± 11.8	51.1 ± 11.5	< 0.001	58.8 ± 10.9	50.6 ± 11.7	< 0.01
BMI*	23.4 ± 3.5	24.5 ± 2.7	0.04	23.1 ± 3.4	25.3 ± 3.5	< 0.01
Distribution			0.09			0.95
unilateral	61 (72.6)	71 (84.5)		30 (78.9)	28 (82.4)	
bilateral	23 (27.4)	13 (15.5)		8 (21.1)	6 (17.6)	
Tumor diameter(mm)*	34.1 ± 16.5	22.2 ± 7.9	< 0.001	38.5 ± 19.8	20.6 ± 6.7	< 0.001
Unenhanced attenuation (HU)*	37.9 ± 6.6	23.2 ± 8.9	< 0.001	39.1 ± 9.1	26.1 ± 11	< 0.001
Radscore^‡^	-1.7(-2.6, -0.8)	2.1(0.6, 2.8)	< 0.001	-2.3(-2.9, -1.0)	1.1(0.2, 2.4)	< 0.001

Except where indicated, data are numbers of patients, with percentages in parentheses. BMI, body mass index; HU, Hounsfield Unit.

*Data are means ± standard deviations.

^‡^Data are median and interquartile range (IQR).

**Table 2:** Comparison of clinical and imaging characteristics between institution 1 and institution 2.

Variable	Institution 1	Institution 2		p-value
Gender (%)				0.90
female	113 (47.1)	24 (46.2)		
male	127 (52.9)	28 (53.8)		
Age*	54.5 ± 11.9	57.8 ± 14.6		0.08
BMI^‡^	23.8 (21.8, 26.2)	23.3 (20.6, 25.7)		0.18
			
Distribution (%)				0.51
unilateral	190 (79.2)	39 (75.0)		
bilateral	50 (20.8)	13 (25.0)		
Tumor diameter(mm)^‡^	24.0 (18.3, 34.0)	27.0 (20.3, 38.8)		0.29
Unenhanced attenuation (HU)^‡^	33.0 (22.0, 40.0)	33.0 (25.0, 37.0)		0.91
Radscore^‡^	-0.3 (-1.8, 2.0)	-0.4 (-1.9, 1.4)		0.70

Except where indicated, data are numbers of patients, with percentages in parentheses. BMI, body mass index; HU, Hounsfield Unit

*Data are means ± standard deviations.

^‡^Data are median and interquartile range (IQR).

**Table 3:** Results of univariate and multivariate analysis for lipid-poor adrenal adenomas in the training set.

	Univariate logistic analysis		Multivariate logistic analysis	
Variable	OR (95% CI)	p-value	OR (95% CI)	p-value
Gender (Male)	0.19 (0.09-0.36)	<0.001	0.21 (0.08-0.56)	<0.01
Age	0.96 (0.93-0.98)	<0.01	0.94 (0.90-0.98)	0.02
BMI	1.11 (1.01-1.22)	0.04	1.01 (0.86-1.19)	0.62
Distribution (unilateral)	0.49 (0.23-1.04)	0.63		
Tumor diameter (per 1 mm)	0.91 (0.88-0.95)	<0.001	0.23 (0.11-0.49)	<0.01
Unenhanced attenuation (per 1 Hu)	0.81 (0.76-0.86)	<0.001	0.86 (0.81-0.91)	<0.001

### Image Characteristics

The mean unenhanced attenuation was lower in the lipid-poor adenomas than in the non-adenomas in all data sets (23.2 ± 8.9 HU vs 37.9 ± 6.6 HU; 26.1 ± 11.0 HU vs 39.1 ± 9.1 HU; 24.9 ± 10.2 HU vs 36.2 ± 6.8 HU; all p < 0.05). The lipid-poor adenomas had smaller diameters than did the non-adenomas (22.2 ± 7.9 mm vs 34.1 ± 16.5 mm; 20.6 ± 6.7 mm vs 38.5 ± 19.8 mm; 21.6 ± 8.0 mm vs 35.0 ± 11.1 mm; all p < 0.05) in all data sets. There was no difference in tumor distribution between lipid-poor adenomas and non-adenomas. There were no significant differences in any of the image characteristics between the training and test sets.

Multivariable logistic regression analysis revealed that unenhanced attenuation and tumor diameter were independent predictors of lipid-poor adenomas (**Table 3**). The results of the interobserver and intraobserver reproducibility of the image features are shown in **Table S2** in the appendix.

### Feature Selection and Radiomic Signature Construction

**Figure 2:**
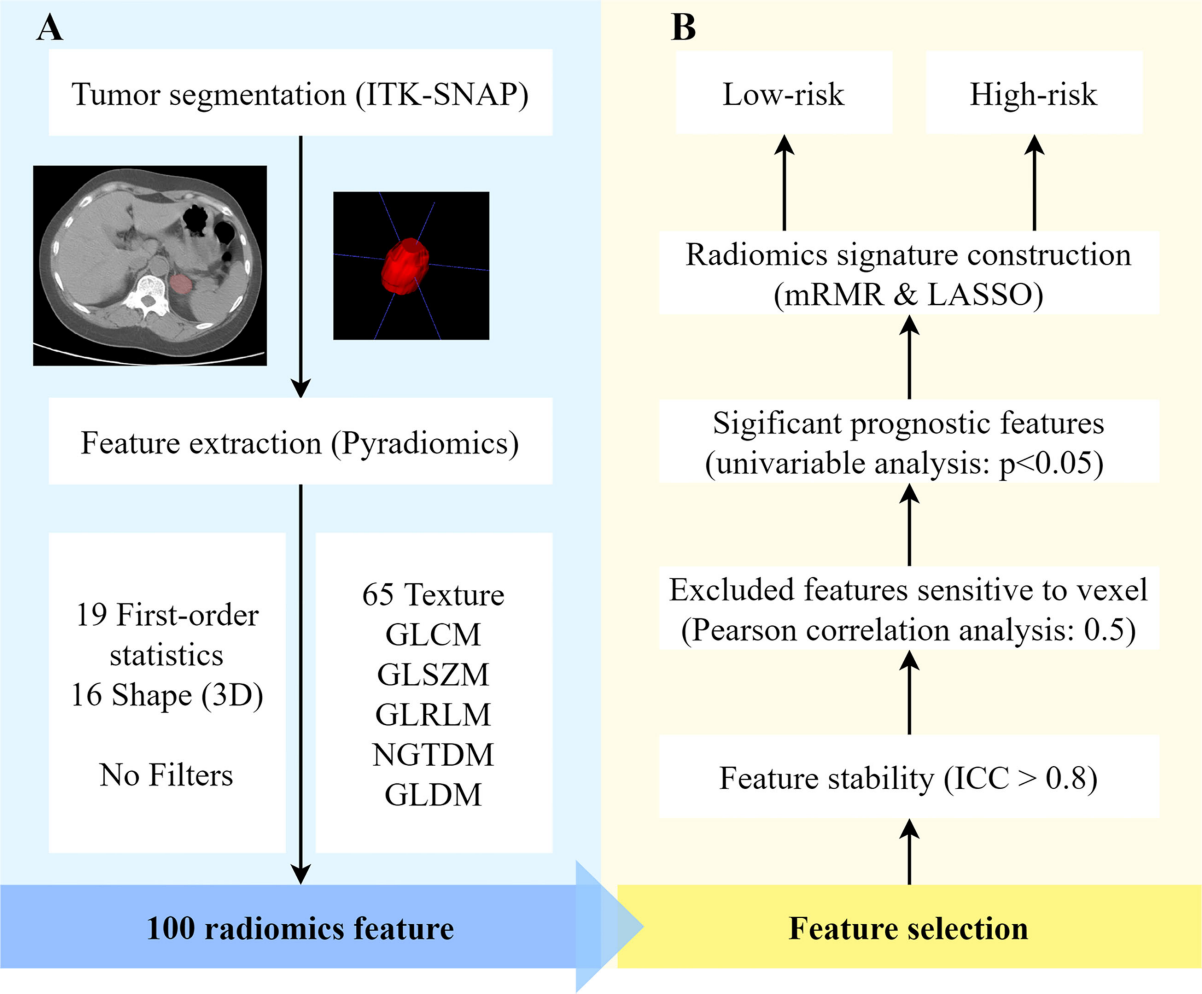
Radiomics process based on adrenal lipid-poor adenomas and nonadenomas. **(A)** Feature extraction and **(B)** feature selection. ICC, intraclass correlation coefficient; LASSO, least absolute shrinkage and selection operator; mRMR, minimal redundancy maximum relevance; ROI, region of interest; 3D, three-dimensional.

**Figure 2** shows the flowchart for obtaining the radiomic signature. One hundred original features were extracted, including 19 histogram features, 16 morphological features, and 65 textural features. Seventy-one features were excluded based on the following criteria: poor reproducibility (n = 52), correlation with the number of voxels (n = 17), and nonsignificance in the univariate analysis (n = 2). Of these features, six related features with nonzero coefficients in the LASSO logistic regression model were obtained from non-enhanced CT images, using the following formula:


rad−score=−0.108∗original_firstorder_Median+13.957∗original_glszm_SizeZoneNonUniformityNormalized−0.052∗original_firstorder_90Percentile−0.642∗original_gldm_DependenceEntropy−0.576∗original_gldm_DependenceVariance−0.167∗original_firstorder_RobustMeanAbsoluteDeviation+7.233

The rad-scores of adrenal lipid-poor adenomas were higher than those of adrenal non-adenomas in all data sets (all p < 0.05). The rad-scores of adrenal lipid-poor adenomas, pheochromocytomas, and metastases are shown in **Figure S1** in the appendix. The results of interobserver and intraobserver reproducibility of radiomics features are shown in **Table S2** in the appendix.

### Comparison of the Diagnostic Performance of the Three Models

**Figure 3:**
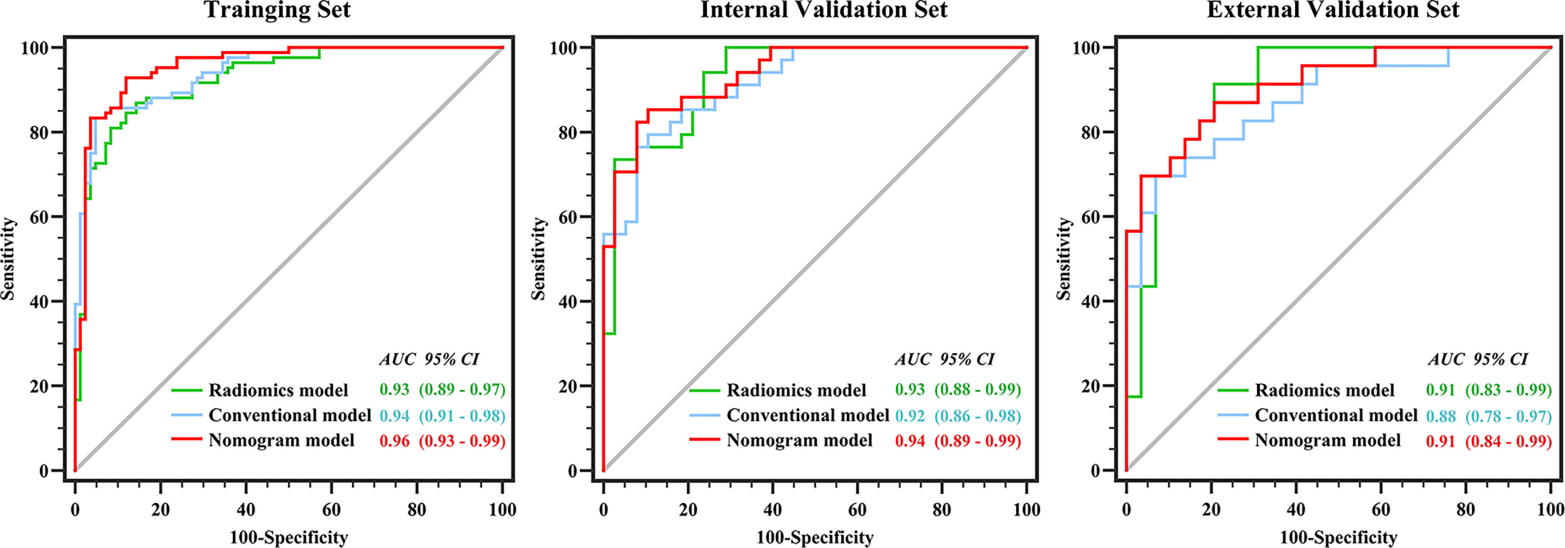
Performance of conventional model, radiomics model and nomogram model in three datasets. There was no difference between the conventional and nomogram or radiomics models in identifying lipid-poor adenomas in any of the datasets (all p > 0.05). The diagnostic performance of the nomogram model was superior to that of the radiomics model only in the training set (p < 0.05).

**Figure 4:**
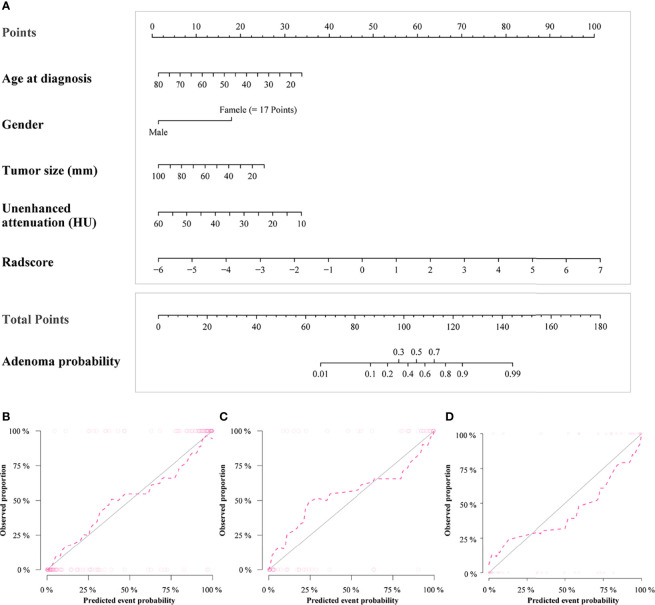
Radiomics nomogram for predicting lipid-poor adenomas **(A)**. Calibration curves of the radiomics nomogram in the training set **(B)**, test set **(C)** and external validation set **(D)**. The calibration curves show calibration of the nomogram in terms of agreement between the predicted risk of lipid-poor adenomas and pathological findings. The closer the dotted line fit to the ideal line, the better the predictive accuracy of the nomogram.

**Figure 3** shows the ROCs of the three models. The AUC of the conventional model, which included sex, age, unenhanced attenuation, and tumor diameter, was 0.94 (95% confidence interval [CI] 0.91−0.98) in the training set and 0.92 (95% CI 0.86−0.98) in the test set. The AUC of the radiomics model was 0.93 (95% CI 0.89−0.97) and 0.93 (95% CI 0.88−0.99) in the training and test sets, respectively. The nomogram model was constructed (**Figure 4A**), and the AUC of the nomogram model was 0.96 (95% CI 0.93−0.99) in the training set and 0.94 (95% CI 0.89−0.99) in the test set). In the external validation set, the AUCs of the conventional model, radiomics model, and nomogram model were 0.88 (95% CI 0.78−0.97), 0.91(95% CI 0.83−0.99), and 0.91 (95% CI 0.84−0.99), respectively.

Detailed information on the performance of the different models is presented in **Table 4**. There was no difference between the conventional and nomogram or radiomics models in identifying lipid-poor adenomas from non-adenomas in any of the datasets (all p > 0.05). The diagnostic performance of the nomogram model was superior to that of the radiomics model only in the training set (p < 0.05). The calibration curve of the nomogram model showed that the model had goodness of fit was good (**Figures 4B–D**). The decision curve showed that the nomogram model would benefit clinicians in the diagnosis of adrenal lipid-poor adenomas (**Figure S2** in the appendix).

**Table 4:** Detailed diagnosis performance of models in all datasets.

	Model	Accuracy (95% CI)	Sensitivity	Specificity	PPV	NPV
Training set	Radiomics	0.86 (0.80-0.91)	0.87	0.86	0.86	0.87
	Conventional	0.89 (0.84-0.94)	0.83	0.95	0.95	0.85
	Nomogram	0.90 (0.85-0.94)	0.93	0.88	0.89	0.93
Test set	Radiomics	0.82 (0.71-0.90)	0.76	0.87	0.84	0.80
	Conventional	0.83 (0.73-0.91)	0.74	0.92	0.89	0.80
	Nomogram	0.88 (0.78-0.94)	0.85	0.89	0.88	0.87
External validation set	Radiomics	0.83 (0.70-0.92)	0.83	0.83	0.79	0.86
	Conventional	0.79 (0.65-0.89)	0.70	0.86	0.80	0.78
	Nomogram	0.77 (0.63-0.87)	0.87	0.69	0.69	0.87

PPV, positive predict value; NPV, negative predict value.

The cutoff of radiomics model is -0.1155177, the cutoff of conventional model is 0.6482431, the cutoff of nomogram model is -0.6291612.

**Figure 5:**
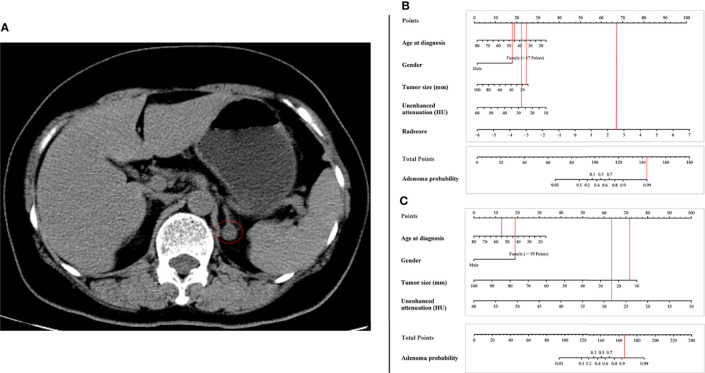
Examples of the nomogram in clinical practice. **(A)** Axial unenhanced abdominal CT images in a 55-year-old woman with adrenal lipid-poor adenoma from external validation set. Figures illustrate the process of calculating the probability of adrenal lipid-poor adenoma using **(B)** radiomics nomogram and **(C)** conventional nomogram. **(B)** CT features were analyzed as follows: tumor diameter = 13 mm, unenhanced attenuation = 28 HU, radscore = 2.67. The total score is 168, which corresponds to an adenoma probability of about 0.99. **(C)** CT features were analyzed as follows: tumor diameter = 13 mm, unenhanced attenuation = 28 HU. The total score is 147, which corresponds to an adenoma probability of greater than 0.9.

Examples of clinical uses of the nomogram are shown in **Figure 5**.

## Discussion

Adrenal lipid-poor adenomas hamper the distinction of adenomas from non-adenomas; yet, this is an important matter as the differential diagnosis affects clinical decisions. To our knowledge, this is the first study to identify adrenal lipid-poor adenomas and non-adenomas using radiomics with a relatively large sample. This study developed an unenhanced CT-based nomogram model that showed excellent diagnostic performance (AUC 0.96, sensitivity, 92.9%; specificity, 88.1%) in distinguishing lipid-poor adenomas, which means that contrast-enhanced CT can be avoided in patients with adrenal lesions. In the process of feature extraction, customized parameters, including bin-width and voxel array shift according to data characteristics, were applied. Feature extraction is a key step in radiomics studies, and it is necessary to consider customizing the extraction rather than using default parameters in the software package in most situations. The radiomics quality score in this study was 21 (**Table S3** in the appendix) (18). Among the six radiomics features, GLDM-dependence variance is a measure of the variance in dependence size, which reflects the inhomogeneity of the texture of the image. First order-90 percentile and first order-median are histogram features that represent the distribution of the intensity of the voxels of the ROI. GLSZM-SizeZoneNonUniformityNormalized reflects the fineness of an image. Our study found that the images of adenomas had a more uniform and smooth texture, while the voxel intensity was lower than that of non-adenomas.

A previous study found that washout CT could effectively identify adrenal adenomas with a sensitivity of 93.9% and specificity of 95.8% (23); however, in their study, adenomas were composed of fatty adenomas and lipid-poor adenomas, which may overestimate the sensitivity because the sensitivity of washout CT in identifying adrenal lipid-rich adenomas was higher than that of lipid-poor adenomas (24). However, washout CT requires a relatively long-delay scanning time, and examination time and radiation exposure are inevitable problems (25). Kumagae et al. reported that shortening the delay in scanning time would reduce the diagnostic performance (26). Our nomogram model was based only on unenhanced CT images, yielded a sensitivity of 92.9% and specificity of 88.1%, and all adrenal adenomas identified were lipid-poor adenomas. The signal of lesions containing fat in the cytoplasm decreases during the inverse phase of chemical-shift imaging. A previous study showed that MRI chemical shift can distinguish adrenal lipid-poor adenomas from non-adenomas, but with inadequate diagnostic performance (sensitivity, 75.7%; specificity, 60%) (15). Ho et al. found that CT-based texture features could distinguish adrenal lipid-poor adenomas from malignant lesions with an AUC of 0.8, even based on their sample size of only 23, which suggests the feasibility of radiomics in prognosing adrenal lipid-poor adenomas (27). Zhang et al. found that lipid-poor adenomas had lower mean gray-level intensity and entropy than pheochromocytomas, which was consistent with our findings (19); however, the AUC of their model was 0.86, and the non-adenomas in our study included various adrenal tumors.

Unexpectedly, the conventional model combined with unenhanced attenuation and tumor diameter also showed good performance in identifying lipid-poor adenomas, with an AUC of 0.94 in the training set and 0.86 in the external validation set. There was no statistically significant difference between the performance of the conventional model and our nomogram model. Adrenal lipid-poor adenomas had lower unenhanced attenuation, while the two radiomics features of the first order-90 percentile and first order-median were negatively correlated with adrenal lipid-poor adenomas, which was mutually validated. Previous studies might have paid more attention to the unenhanced CT attenuation value of 10 HU as a cutoff point; nevertheless, unenhanced attenuation demonstrates good ability to identify adrenal lipid-poor adenomas, although it was not further explored in some studies (28, 29). In the training group of our study, the conventional model misjudged six adenomas and one non-adenomas, while the radiomics model judged all cases correctly. Further, in the training group, the radiomics model misjudged three adenomas and nine non-adenomas, while the conventional model judged all cases correctly. The radiomics features and conventional imaging features may manifest the characteristics of adrenal lipid-poor adenomas from different angles, and they should be complementary in distinguishing adrenal adenomas from non-adenomas.

Our study has some limitations. First, the number of samples of some types of non-adenomas with low incidence (i.e., gangliocytoma and lymphoma) was insufficient, and the ability of the model to differentiate them was uncertain (30). Second, only 3D ROIs were applied; although previous studies have shown that the ability of 3D ROIs to reflect the heterogeneity of tumors was better than that of 2D ROIs (31), 2D ROIs have the advantage of more convenient operation and may therefore be more feasible to use. Third, the contrast-enhanced CT examination in this study was only used to ensure the accuracy of the measurement of unenhanced attenuation. Contrast-enhanced CT may contain more information about adrenal lesions, and the diagnostic performance of contrast-enhanced CT-based radiomics models is unknown. In addition, the different doses of the contrast agent and scanning parameters of each institution may have a significant impact on the contrast-enhanced image (32).

## Conclusion

In this study, we developed a CT-based radiomics nomogram model, which could effectively identify adrenal lipid-poor adenomas and non-adenomas as a novel, non-invasive method for assisting clinical decision-making. In addition, our study implied that the diagnostic power of conventional unenhanced CT imaging features for identifying adrenal adenomas may be underestimated, and further exploration is worthy.

## Data Availability Statement

The original contributions presented in the study are included in the article/**Supplementary Material**. Further inquiries can be directed to the corresponding author.

## Author Contributions

BZ, HZ, WP, and XD: conception and design. HZ, XL, JC, SJ, and WJ: collection and assembly of data. HZ, and BZ: data analysis and interpretation. BZ, XD, and JY: manuscript writing. All authors contributed to manuscript revision, read, and approved the submitted version.

## Conflict of Interest

The authors declare that the research was conducted in the absence of any commercial or financial relationships that could be construed as a potential conflict of interest.

## Publisher’s Note

All claims expressed in this article are solely those of the authors and do not necessarily represent those of their affiliated organizations, or those of the publisher, the editors and the reviewers. Any product that may be evaluated in this article, or claim that may be made by its manufacturer, is not guaranteed or endorsed by the publisher.

## Abbreviations

CT, computed tomography; AUC, area under the receiver operating characteristic curve; CI, confidence interval; ROI, region of interest; MRI, magnetic resonance imaging; ROC, receive operating characteristic; 18 FDG, 18F-fluorodeoxyglucose; VIF, variance inflation factor.
