# Supplementary material for: Can Radiomics Provide Additional Diagnostic Value for Identifying Adrenal Lipid-Poor Adenomas From Non-Adenomas on Unenhanced CT?
Source: Front Oncol. 2022 Apr 29;12:888778. doi: 10.3389/fonc.2022.888778 (PMC9102986; doi:10.3389/fonc.2022.888778)
Supplement: Supplementary file 1 [file DataSheet_1.docx]

**The detailed scanner parameters:**

The CT protocols for the Discovery CT750 HD scanner were as follows: collimation, 64 × 0.625 mm; rotation time, 0.6 seconds; pitch factor, 0.984; 120 kVp, 180–300 mAs with automatic tube current modulation. The CT protocols for the uCT 530 scanner were as follows: collimation, 40 × 0.55 mm; rotation time, 0.8 s; pitch factor, 1.075; 120 kVp, 180–300 mAs with automatic tube current modulation. The CT protocols for the BrightSpeed 16 scanner were as follows: collimation, 16 × 0.625 mm; rotation time, 0.5 s; pitch factor, 1.075; 120 kVp, 180–300 mAs with automatic tube current modulation. Each patient received 300 or 350 mgI/mL of contrast material (iohexol or iodopanol) inserted into the anterior elbow veins, with a total iodine dose of 400 mgI/kg. By using automatic injection tracking technology with automatic scanning trigger software, the portal venous phase image was obtained 45 s after the enhancement reached the trigger threshold of 100 HU at the level of the abdominal aorta.

**Pyradiomics setting parameters:**

**imageType:**

**Original: {}**

**featureClass:**

**shape:**

**firstorder:**

**glcm:**

**- 'Autocorrelation' - 'JointAverage' - 'ClusterProminence' - 'ClusterShade' - 'ClusterTendency' - 'Contrast' - 'Correlation' - 'DifferenceAverage' - 'DifferenceEntropy' - 'DifferenceVariance' - 'JointEnergy' - 'JointEntropy' - 'Imc1' - 'Imc2' - 'Idm' - 'Idmn' - 'Id' - 'Idn' - 'InverseVariance' - 'MaximumProbability' - 'SumEntropy' - 'SumSquares'**

**glrlm:**

**glszm:**

**gldm:**

**setting:**

**# Normalization:**

**# normalize: true**

**# normalizeScale: 500**

**# Resampling:**

**interpolator: 'sitkBSpline'**

**resampledPixelSpacing: [1, 1, 1]**

**padDistance: 10**

**# Image discretization:**

**binWidth: 3**

**# first order specific settings:**

**voxelArrayShift: 450**

**# Misc:**

**# default label value. Labels can also be defined in the call to featureextractor.execute, as a commandline argument,**

**# or in a column "Label" in the input csv (batchprocessing)**

**label: 1**

FigureS1


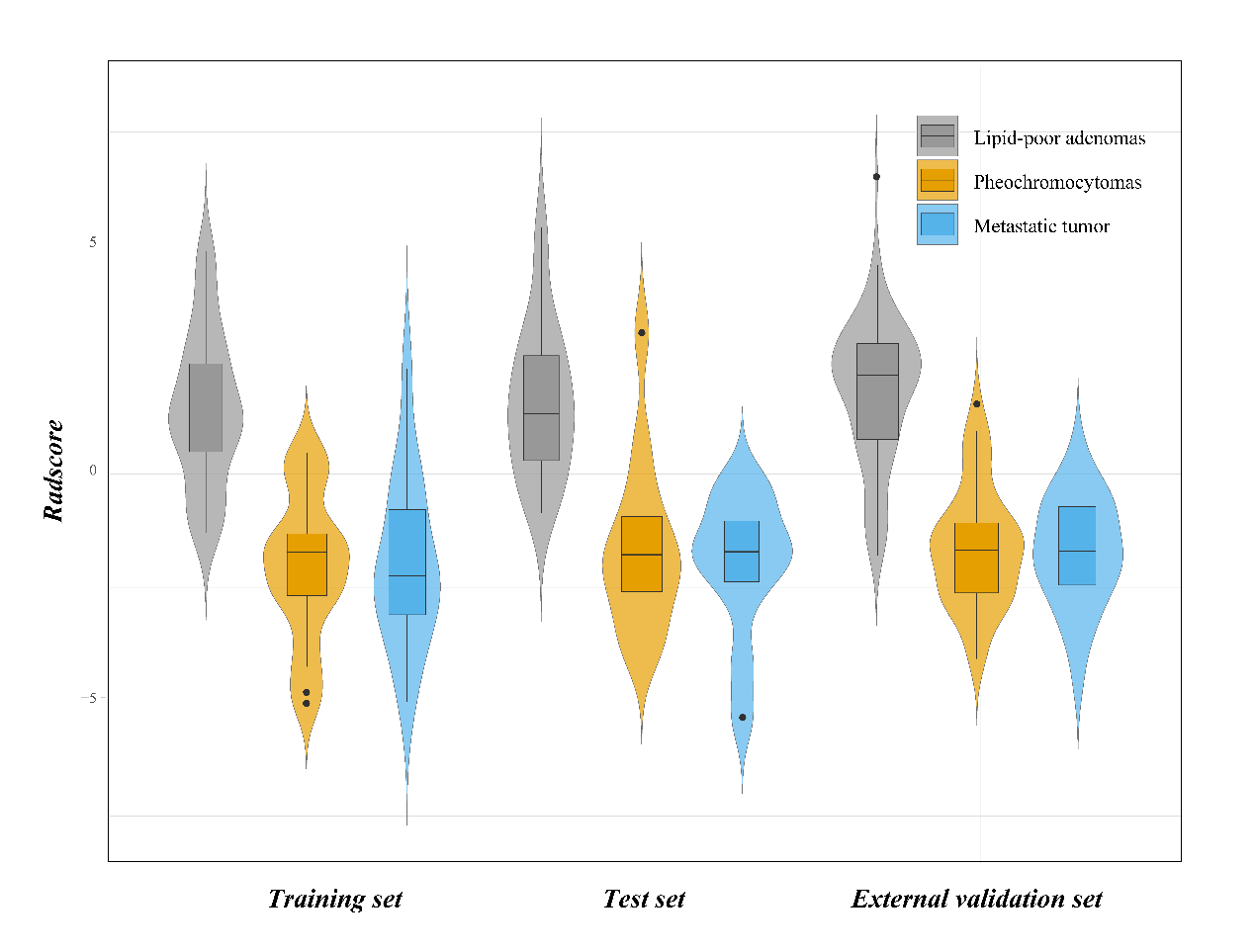
The rad-scores of adrenal lipid-poor adenomas, pheochromocytomas and metastases in three datasets. There was statistical difference between lipid-poor adenomas and pheochromocytomas as well as metastases in all datasets.

FigureS2


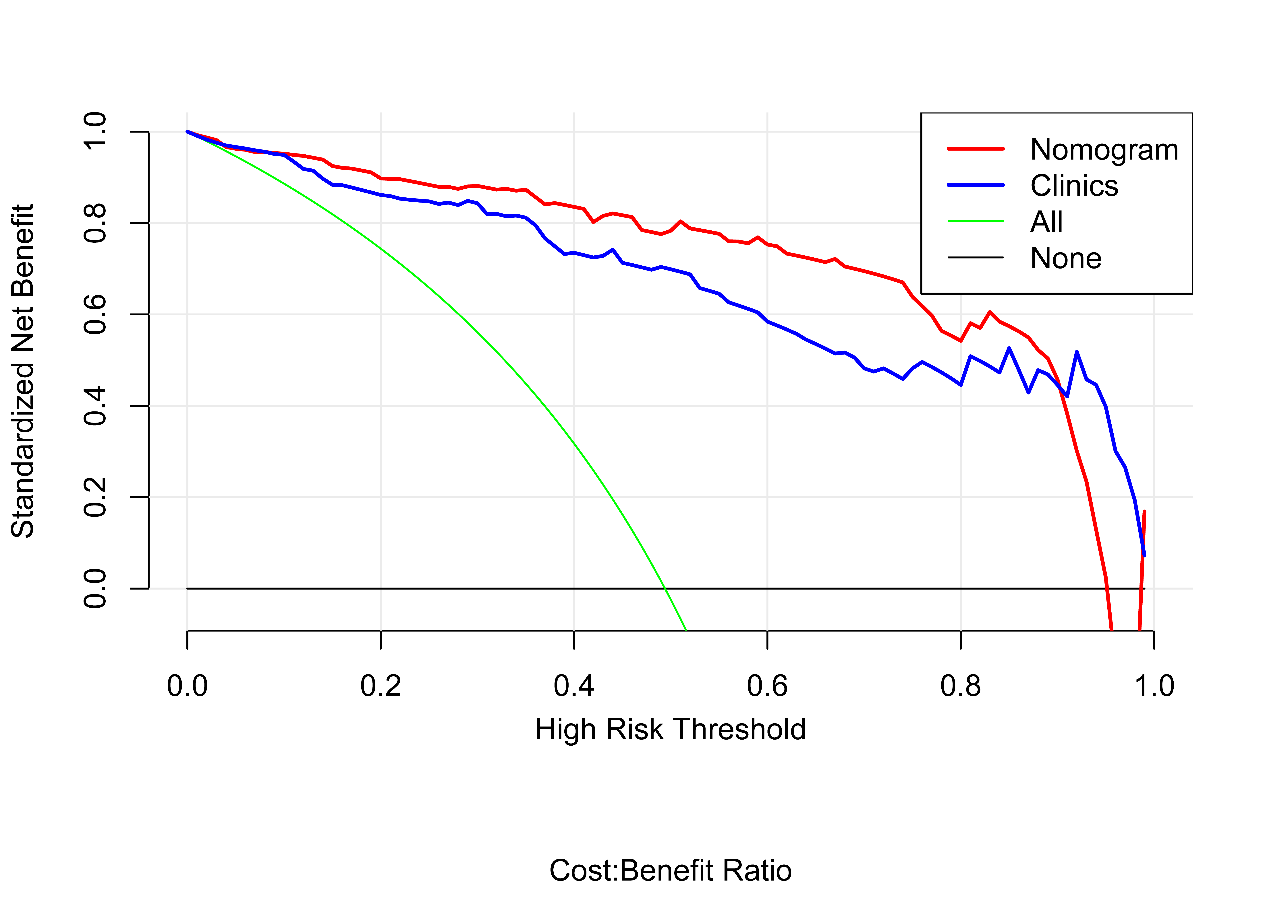
 DCA for the radiomics nomogram. The y-axis shows the net benefit. The pink line represents the radiomics nomogram. The green line indicates the hypothesis that all patients had adrenal lipid-poor adenomas. The black line represents the hypothesis that no patients had adrenal lipid-poor adenomas. The x-axis shows the threshold probability, which is where the expected benefit of treatment is equal to the expected benefit of not undergoing treatment. For example, if the possibility of adrenal lipid-poor adenomas is over the threshold probability, then the treatment strategy for adrenal lipid-poor adenomas should be adopted. The decision curves indicate that, when the threshold probability is between 0.1 and 0.9, using the radiomics nomogram to predict adrenal lipid-poor adenomas adds more benefit than treating either all or no patients.

**Table S1** Clinical and imaging characteristics of training and test sets

| Variable | Training set  (n = 168) | Test set  (n =72) | p-value |
| --- | --- | --- | --- |
| Gender (%) |  |  | 0.25 |
| female | 75 (44.6) | 38 (52.8) |  |
| male | 93 (55.4) | 34 (47.2) |  |
| Age* | 54.0 (47.0, 63.0) | 56.5 (46.0, 64.0) | 0.57 |
| BMI* | 23.8 (22.0, 26.0) | 23.8 (21.3, 26.5) | 0.95 |
| Distribution (%) |  |  | 0.73 |
| unilateral | 132 (78.6) | 58 (80.6) |  |
| bilateral | 36 (21.4) | 14 (19.4) |  |
| Tumor | 24.0 (19.0,34.0) | 25.0 (18.0-35.5) | 0.72 |
| diameter(mm)* |  |  |  |
| Unenhanced attenuation (HU) * | 32.0 (21.0,38.0) | 34.0 (24.0, 41.5) | 0.14 |
| Radscore* | -0.1 (-1.8, 2.1) | -0.5 (-2.3, 1.1) | 0.15 |

Note.—Except where indicated, data are numbers of patients, with percentages in parentheses. BMI = body mass index, HU = Hounsfield Unit.

* Data are median and interquartile range (IQR).

**Table S2** **Introbserver and interobserver agreement of tradition image features and radiomics signatures**

| Variables | Interobserver (95% CI) | Intraobserver (95% CI) |
| --- | --- | --- |
| Tumor diameter | 0.966 (0.936-0.983) | 0.962 (0.927-0.980) |
| Unenhanced attenuation | 0.987 (0.975-0.993) | 0.991 (0.983-0.995) |
| Original_firstorder_Median | 0.979 (0.960-0.989) | 0.983 (0.965-0.991) |
| Original_glszm_SizeZone NonUniformityNormalized | 0.879 (0.799-0.942) | 0.894 (0.805-0.944) |
| Original_firstorder_ 90Percentile | 0.984 (0.969-0.992) | 0.991 (0.982-0.995) |
| Original_gldm_ DependenceEntropy | 0.843 (0.718-0.916) | 0.902 (0.819-0.948) |
| Original_glrlm_RunVariance | 0.973 (0.948-0.986) | 0.987 (0.975-0.993) |

ICCs: intraclass correlation coefficients

**Table S3 Elements of the RQS and rating achieved by this study.**

| RQS scoring item | Description | Score |
| --- | --- | --- |
| Image Protocol | + 1 for well documented protocols, + 1 for publicly available protocols | 1 |
| Multiple Segmentations | + 1 if segmented multiple times (different physicians, algorithms, or perturbation of regions of interest) | 1 |
| Phantom Study | + 1 if texture phantoms were used for feature robustness assessment | 0 |
| Multiple Time Points | + 1 multiple time points for feature robustness assessment | 0 |
| Feature Reduction | − 3 if nothing, + 3 if either feature reduction or correction for multiple testing | 3 |
| Non Radiomics | + 1 if multivariable analysis with non-radiomics features | 1 |
| Biological Correlates | + 1 if present | 1 |
| Cut-off | + 1 if cutoff either pre-defined or at median or continuous risk variable reported | 1 |
| Discrimination  and Resampling | + 1 for discrimination statistic and statistical significance, + 1 if resampling applied | 2 |
| Calibration | + 1 for calibration statistic and statistical significance, +1 if resampling applied | 2 |
| Prospective | + 7 for prospective validation within a registered study | 0 |
| Validation | − 5 if no validation/+ 2 for internal validation/+ 3 for external validation/+ 4 two external validation datasets or validation of previously published signature /+ 5 validation on ≥ 3 datasets from > 1 institute | 3 |
| Gold Standard | + 2 for comparison to gold standard | 2 |
| Clinical Utility | + 2 for reporting potential clinical utility | 2 |
| Cost-effectiveness | + 1 for cost-effectiveness analysis | 0 |
| Open Science | + 1 for open-source scans, + 1 for open-source segmentations, + 1 for open-source code, + 1 open-source，representative segmentations and features | 2 |
| RQS: Radiomics Quality Score | | |
